# Air Pollution and Otitis Media in Children: A Systematic Review of Literature

**DOI:** 10.3390/ijerph15020257

**Published:** 2018-02-03

**Authors:** Gayan Bowatte, Rachel Tham, Jennifer L. Perret, Michael S. Bloom, Guanghui Dong, Nilakshi Waidyatillake, Dinh Bui, Geoffrey G. Morgan, Bin Jalaludin, Caroline J. Lodge, Shyamali C. Dharmage

**Affiliations:** 1 Allergy and Lung Health Unit, Centre for Epidemiology and Biostatistics, School of Population & Global Health, The University of Melbourne, Melbourne, VIC 3010, Australia; rtham@student.unimelb.edu.au (R.T.); jennifer.perret@unimelb.edu.au (J.L.P.); nilakshi.waidyatillake@unimelb.edu.au (N.W.); d.bui2@student.unimelb.edu.au (D.B.); clodge@unimelb.edu.au (C.J.L.); s.dharmage@unimelb.edu.au (S.C.D.); 2 National Institute of Fundamental Studies, Kandy 20000, Sri Lanka; 3 Department of Environmental Health Sciences School of Public Health University at Albany, State University of New York, Rensselaer, NY 12144, USA; mbloom@albany.edu; 4 Department of Epidemiology and Biostatistics School of Public Health University at Albany, State University of New York, Rensselaer, NY 12144, USA; 5 Guangzhou Key Laboratory of Environmental Pollution and Health Risk Assessment, Department of Preventive Medicine, School of Public Health, Sun Yat-Sen University, Guangzhou 510080, China; donggh5@mail.sysu.edu.cn; 6 University Centre for Rural Health, School of Public Health, University of Sydney, Sydney, NSW 2480, Australia; geoffrey.morgan@sydney.edu.au; 7 Healthy People and Places Unit, South Western Sydney Local Health District, Liverpool, NSW 2170, Australia; b.jalaludin@unsw.edu.au

**Keywords:** middle ear infection, otitis media, air pollution

## Abstract

Young children are particularly vulnerable to otitis media (OM) which globally affects over 80% of children below the age of 3 years. Although there is convincing evidence for an association between environmental tobacco smoke exposure and OM in children, the relationship with ambient air pollution is not clear. We aimed to systematically review the literature on the relationship between ambient air pollution exposure and OM in children. A systematic search was performed in PubMed and EMBASE databases. Of 934 references identified, 24 articles were included. There is an increasing body of evidence supporting an association between higher ambient air pollution exposure and a higher risk of OM in children. While NO_2_ showed the most consistent association with OM, other specific pollutants showed inconsistent associations. Studies were mainly conducted in high/middle income countries with limited evidence from low-income countries. Although there was a general consensus that higher air pollution exposure is associated with a greater prevalence of OM, the evidence for associations with specific pollutants is inconsistent. More well-designed studies on associations between specific air pollutants as risk factors for OM are warranted, especially in low income countries with high air pollution levels.

## 1. Introduction

Middle ear inflammation, commonly known as otitis media (OM), is a multifactorial disease of the middle ear, with a high prevalence among young children [1]. Acute OM (AOM) is characterized by infection of the middle ear with associated symptoms. OM with effusion (OME) is characterized by middle ear effusion and inflammation without infection. Chronic Suppurative OM (CSOM) presents as purulent otorrhea associated with perforation of the tympanic membrane [2]. 

Young children are particularly vulnerable to OM due to their smaller and shorter middle ear anatomical features than in adults, and more horizontally aligned Eustachian tubes, and frequent upper respiratory tract infections [3]. The sequelae of OM include hearing loss, language delay, impaired cognitive development and social difficulties in later life [4]. Globally, OM affects over 80% of children aged below 3 years. Many of them, 30–45%, have suffered two or more episodes [5]. The global estimated annual incidence of AOM and CSOM is 740 million combined, with nearly 21,000 deaths attributable to complications from OM, such as meningitis or brain abscess [6]. OM is the leading cause of antibiotic use and impaired quality of life in children. They pose significant economic and social burdens on families and the health care system [7,8].

The upper respiratory tract, including nose and upper airway, plays an important role in filtering and conditioning inspired air. The nose and mouth are the main entry points for inspired air and are connected via the nasopharynx. The nasopharynx connects to the middle ear via the Eustachian tube, located at the back of the nasopharynx. This direct connection to the middle ear creates a link between inspired air and the middle ear. Larger airborne particles, (e.g., particulate matter less than 10 µm in diameter (PM_10_) and some liquid pollutants, (e.g., formaldehydes), are dissolved or otherwise trapped by the nasal mucosa and transported to the back of the nasopharynx, where they are either swallowed or expectorated [9]. However, other pollutants in air, such as, gases and particles less than 2.5 µm in diameter (PM_2.5_) may pass through the nasopharyngeal cavity and enter the airways and lungs. Given the direct connection between the nasopharynx and middle ear, these pollutants may interact with the Eustachian tube epithelium. This epithelium includes columnar ciliated cells with motile, hair like appendages called cilia that beat rhythmically in the direction of the nasopharynx and are involved in mucociliary clearance and drainage of middle ear fluid. The Eustachian tube also allows air exchange and pressure balance. When inflammatory agents such as viral or bacterial pathogens, allergens, pollutants and other irritants interact with the nasal mucosa, resulting inflammation can narrow or block the Eustachian tube. Dysfunction in the Eustachian tube can lead to middle ear fluid stasis and subsequent middle ear infection.

Exposure to environmental tobacco smoke especially from parents who smoke in the home environment and the development of OM in children is well established. A systematic review by Jones et al. reported in 2012 that living with a smoker increased the risk of children’s OM by 62% [10]. The effect of long term exposure to environmental tobacco smoke on histological changes in middle ear and Eustachian tube mucosa have been demonstrated in animal studies [11]. Although, there is convincing evidence for an association between environmental tobacco smoke exposure and OM in children, the relationship with ambient air pollution exposure is not yet established. The last review on this topic, published in 2004 by Heinrich et al., could not reach conclusions on this relationship due to the lack of well-designed studies [12]. Since then a considerable number of published studies have been added to the body of literature on air pollution and OM, including large scale epidemiological studies [13,14] and studies investigating plausible mechanisms using state of the art molecular techniques [15,16]. 

There is growing evidence that air pollution exposure is associated with asthma and allergies in children [17]. Air pollutants such as particulate matter (PM), trigger oxidative responses and inflammation in lung epithelium leading to asthma and allergies. Animal studies provide evidence that air pollutants, such as sulphur dioxide (SO_2_), impair the mucociliary function of the Eustachian tube and increase middle ear mucus secretion [18]. Similarly, evidence from epidemiological studies supports a link between air pollution and OM; however, there has been no recent comprehensive systematic review of these investigations in children. Therefore, we aimed to systematically review studies investigating both short and long-term exposure to ambient air pollution and their relationship with OM in children, to assess the weight of evidence for causality.

## 2. Methods

### 2.1. Search Strategy

We conducted a systematic literature search using PubMed and EMBASE databases, from the time of their inception to 28 October 2017, to capture all studies investigating the effects of air pollution on OM, without restrictions. We included search terms (“Otitis Media” [MeSH Terms]) OR (“Otitis media”) OR (“Middle ear infection”) OR (“ear infection”) AND (“air pollution”) OR (“air quality”) OR (“vehicle emissions”). In addition to our initial electronic search, we manually searched the reference lists of selected papers and important reviews for additional studies of relevance. Gayan Bowatte and Rachel Tham independently reviewed the titles and abstracts of all identified citations for eligibility. A third author was consulted (Shyamali C. Dharmage) in case of disagreement. 

### 2.2. Eligibility Criteria

We retained only English language full text articles describing cohort, case control, case-crossover, cross sectional and time series epidemiologic study designs. The study population of interest was children aged from birth up to 18 years. Our endpoint of interest was otitis media, captured as parent-reported, physician/doctor diagnosed or a hospital diagnosis of middle ear infection, OM, AOM, OME, CSOM or glue ear. Our exposure of interest was air pollution, which included indoor (heating and cooking), industrial, outdoor or vehicle emission related. To focus on the impact of ambient air pollutants, we excluded studies of environmental tobacco smoke exposure and OM. 

### 2.3. Quality Assessment/Risk of Bias in Included Studies

Gayan Bowatte and Rachel Tham independently assessed the quality of the selected studies. The Newcastle–Ottawa Scale (NOS) was used for cohort, case control, case-crossover and cross sectional studies [19]. A modified version of a validated quality assessment framework by Zaza et al. [20] to assess and rate the design, validity and reliability, generalisability, risk of bias and reporting was used for time series studies. This method has been used in previous systematic reviews [21,22]. 

The NOS assessed studies were rated as follows: Cohort studies: very good = 9–10; good = 7–8; satisfactory = 5–6; unsatisfactory = 0–4. Cross sectional: very good = 6–7; good = 5; satisfactory = 4; unsatisfactory = 0–3. Time series studies were scored out of 23, with score >17 being categorised as “high” quality. Any disagreements were resolved by consulting a third author (Shyamali C. Dharmage).

### 2.4. Data Extraction

Initial data extraction was done by Gayan Bowatte, then checked independently by Rachel Tham. Data were extracted for the following domains: study setting, study design, age of children, exposure age, outcome age, sample size, definitions of exposure and outcome, main results, and confounders included in analysis.

This review is reported in accordance with the recommendations set forth by the Preferred Reporting Items for Systematic Reviews and Meta-Analyses (PRISMA) statement and was prospectively registered in PROSPERO systematic review registry (registration number—CRD42017082688).

## 3. Results

### 3.1. Search Results

**Figure 1:**
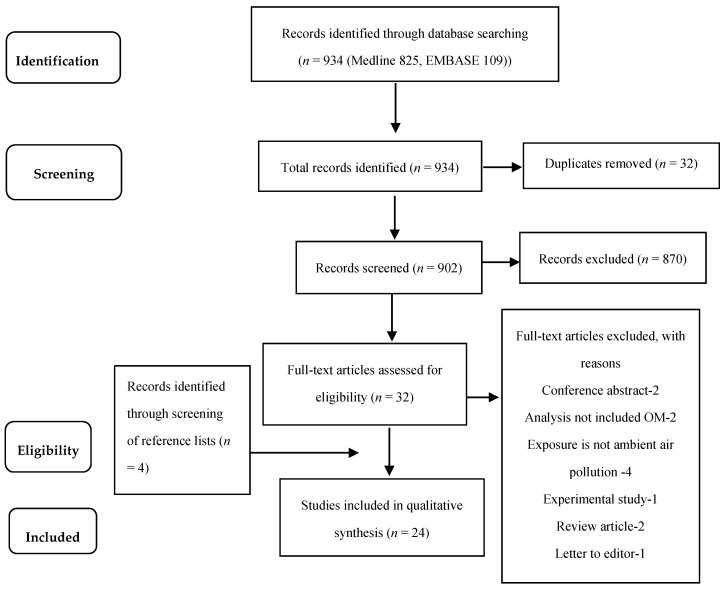
The PRISMA flow diagram for selection of studies for the review. (NOTE: “Records” include a mixture of full journal articles of original research, published abstracts, protocols, invited commentaries, and reviews).

Our electronic literature search found 934 references. After removing 32 duplicates, 902 titles and abstracts were screened. From these, 870 were excluded for failing to meet the eligibility criteria. The majority of the excluded records did not include either an ambient air pollution exposure or an outcome of OM, and some were not in English or were not original research. After reading 32 full text articles, 12 were excluded as: conference abstracts; not OM; not ambient air pollution; experimental studies; and review articles. An additional four studies found through examining the reference lists of original articles selected for full text reading and review papers met inclusion criteria. In total, 24 papers were included in this systematic review (Figure 1).

### 3.2. Characteristics of Included Studies

The 24 included papers represented a range of study designs: nine cohort studies, two case control studies, four case cross-over studies, eight cross sectional studies, and one time-series study (Table 1, Table 2 and Table 3). Among the nine papers describing cohort studies, seven were birth cohorts [13,14,23,24,25,26,27]. Of these, a single paper combined 10 birth cohorts and provided pooled estimates for the risk of air pollution exposure and reported OM [13]. This meta-analysis included some of the birth cohort studies previously published as independent articles and included in our 24 selected full papers. Altogether, there were 15 different cohorts included from the seven papers reporting associations between air pollution and OM from birth cohort studies [13,23,24,25,26,27,28]. All the birth cohorts were from either Europe or North America [13,23,24,25,26,27,28]. The remaining two cohort papers were from a retrospective study of Chinese kindergarten children [29]. The two case control studies were from the United States and Mozambique [30,31]. Of the eight cross sectional studies, seven were from Europe or North America [32,33,34,35,36,37,38] and one was from South America [39]. The case cross-over and time-series studies were from Europe or North America [40,41,42,43,44].

### 3.3. Long-Term Studies

#### 3.3.1. Cohort Studies

All cohort studies were of good to very good quality, with NOS scores ranging from 6 to 9 (Table S1). There was considerable heterogeneity in exposure assessment strategies, life stage of exposure assignment (e.g., in utero, infancy, etc.), outcome assessments and follow up time. 

##### Birth Cohort Studies of Outdoor Air Pollution Exposure and OM

Of the 15 birth cohort studies 11 used land use regression (LUR) models to assign various outdoor pollutants [13,14,23,24], namely nitrogen dioxide (NO_2_), oxides of nitrogen (NO_X_), PM_2.5_, PM_2.5_ absorbance, PM_10_, and coarse particles (PM_2.5–10_). Studies that used LUR to measure air pollution assigned exposure during pregnancy [23], at birth [13,14,24] or during the first year of life [23]. The timing of outcome assessment for these four studies was at the end of two years [13,14,24] or the first 12–18 months [23]. These studies defined OM using diagnostic codes hospital databases recorded at outpatient visits [14] or parent reported doctor diagnosed OM [13,23,24]. For these four papers, sample sizes varied from *n* = 2199 to *n* = 45,513. 

The most comprehensive study from the birth cohort papers combined 10 European general population based birth cohorts to form the European Study of Cohorts for Air Pollution Effects (ESCAPE) Project [13]. The ESCAPE includes cohorts from Sweden, Italy, Germany, Spain, Britain and the Netherlands. The pooled analysis found a significant association between NO_2_ exposure at birth addresses and OM in children during the first two years of life (Odds Ratio (OR) 1.09; 95% CI: 1.02, 1.16 per 10-μgm^−3^ increase). NO_X_ exposure was also found to increase the risk of OM (OR 1.05; 95% CI: 0.98, 1.12 per 10-μgm^−3^ increase). In the same paper, PM_2.5_ data were available only for seven birth cohorts, and pooled estimates showed no significant association with PM_2.5_ exposure at birth address and OM in children (OR 1.06; 95% CI: 0.75, 1.49 per 5-μgm^−3^ increase). Other pollutants, PM_10_, PM_2.5_ absorbance and PM_2.5–10_ reported similar associations [13]. Of the 10 birth cohort studies included in this meta-analysis only two studies (INfancia y Medio Ambiente (INMA)-Gipuzkoa and The Prevention and Incidence of Asthma and Mite Allergy (PIAMA)) showed significant associations between NO_2_ or NO_X_ exposure and OM. Other studies showed no association. The authors have further compared exposure at birth address and OM in the 1st and 2nd years of life separately, finding a higher risk of OM during the first year of life compared to null findings in the second year of life, especially in relation to NO_2_ or PM_2.5_ absorbance exposure. Of these 10 birth cohorts included in ESCAPE, four were from the same INMA cohort, a multi-city study, conducted in four different cities in Spain. Although these cohorts were from the same country they showed heterogeneous results. Similar results were shown in the two German cohorts of GINI and LISA from north and south of Munich. 

The full INMA birth cohort study, in an independent paper, investigated the relationship between NO_2_ or benzene exposure during prenatal and postnatal exposure and OM in children during their first 12–18 months. The researchers found that exposure to NO_2_ during the entire prenatal period, 1st, 2nd, 3rd trimester and first year of life to be associated with increased risk of OM (OR 1.18; 95% CI: 0.98, 1.41; OR 1.11; 95% CI: 0.99, 1.24; OR 1.16; 95% CI: 0.98, 1.37; OR 1.12; 95% CI: 0.98, 1.29; and OR 1.15; 95% CI: 1.01, 1.31; per 10 μgm^−3^ increase, respectively). Similar associations were found with benzene exposure [23]. 

Brauer et al. used LUR to investigated associations between NO_2_, PM_2.5_ and black carbon exposure with OM in children in the Dutch PIAMA and German GINI birth cohorts [24]. Exposure to NO_2_ was associated with an increased risk of OM in the Netherlands, during the first year (OR 1.17; 95% CI: 1.03, 1.34: per 3 μgm^−3^ increase) and first two years (OR 1.14; 95% CI: 1.03, 1.27) of life. PM_2.5_ and that black carbon exposures were associated with increased risk of OM during the first two years of life (OR 1.13; 95% CI: 1.00, 1.27: per 10 μgm^−3^ and OR 1.10; 95% CI: 1.00, 1.22: per 0.5 μgm^−3^, respectively). However, no statistically significant associations were detected in the GINI cohort [24].

MacIntyre et al. studied 45,513 children (76% of all births) in British Colombia, Canada, for associations between air pollution exposure and OM during the first two years of life using LUR [14]. The authors reported statistically significant associations for exposure to higher NO and outdoor wood smoke levels with increased risks for OM (OR 1.10 95% CI 1.07, 1.12: per 24.1 µgm^−3^ and OR 1.32; 95% CI: 1.27, 1.36: per 16 days increase, respectively). However, they found no clear associations for NO_2_, PM_2.5_ or black carbon [14].

A birth cohort from the Czech Republic compared the rates of OM in children living in polluted and non-polluted areas [25]. They reported that children living in the urban and industrial environments had a higher incidence of OM compared to children living in the rural districts (Risk Ratio (RR) 2.3; 95% CI: 1.7, 4.1) [25] (Table 1).

##### Birth Cohort Studies of Indoor Air Pollution Exposure and OM

A birth cohort study from Poland used personal air monitoring in 2nd trimester pregnant women to monitor polycyclic aromatic hydrocarbon (PAH) exposure [26]. They reported associations between PAH exposure and an increased risk of more frequent OM, of longer duration, during the first year of life [26]. 

A Virginia (U.S.) birth cohort study investigating indoor heating and OM during the first year of life, reported that fireplace use or use of a wood heater increased the unadjusted risk of middle year infection during the first year of life. However, these associations were not statistically significant after adjustment for confounders, including heating season [27] (Table 1).

##### Other Cohort Studies of Outdoor and Indoor Air Pollution

In two published papers using the same retrospective cohort of Chinese children aged 3–4 years, Deng et al. investigated the associations between exposure to both outdoor and indoor air pollution by home renovation activities on OM in children. They reported that prenatal SO_2_ exposure estimated from fixed monitoring stations was associated with increased risk of OM (OR 1.44; 95% CI: 1.09, 1.88; per 27 µgm^−3^ increase). Furthermore, the authors detected statistically significant associations between higher SO_2_ exposure during each trimester of pregnancy, and OM at 3 to 4 years of age [45]. Postnatal exposure to new furniture, and household redecoration were also associated with statistically significant higher odds for OM (OR 1.62; 95% CI: 1.05, 2.49 and OR 1.81; 95% CI: 1.12, 2.91, respectively) [29] (Table 1). Summary results of cohort studies for NO_2_, PM_2.5_, PM_2.5_ absorbance and PM_10_ are given in Table S2.

### 3.4. Studies Investigating Lag Effects of Air Pollution Exposure and OM

#### 3.4.1. Case-Crossover Studies of Outdoor Air Pollution Exposure

The quality of cross-over studies varied from 7–9 (Table S3). Of the four case-crossover studies, two investigated associations between ambient outdoor air pollution and OM in children aged ≤18 years, using data from the same U.S. hospitals and defined using the same ICD-9 codes [42,43]. However, the air pollutant exposure assessment strategies differed. Strickland et al. estimated ambient outdoor PM_2.5_ using satellite based Aerosol Optical Depth, from 2002–2010 [42]. The authors reported higher odds for an OM hospital emergency department (ED) visit in association with higher same-day (i.e., Lag 0) PM_2.5_ exposure (Lag 0 OR 1.005; 95% CI: 0.996, 1.014), in a time-stratified case-crossover model [42]. In a U.S. study, Xiao et al. [43], used the EPA Community Multiscale Air Quality (CMAQ) modelling system to assign outdoor ambient air pollution exposures. They found that the joint effect of higher CO, NO_2_, elemental carbon and organic carbon, was associated with a higher risk of ED visits for OM, during 2002 to 2008 (OR 1.018; 95% CI: 1.010, 1.026) [43]. A Canadian case-crossover study, found that each unit increase in the Air Quality Health Index was associated with 5% to 6% increased ED visits for OM three days’ post exposure, in children ≤3 years [41]. The AQHI incorporates ambient O_3_, NO_2_ and PM_2.5_ concentrations into a summary measure for informing the general public about air quality. In another Canadian study, Zemek et al. reported associations between exposure to NO_2_ (lag 2 and 3 days), PM_10_ (lag days 2 and 4), and O_3_ (lag 1 day) and increased ED visits for OM, especially in warm months [44] (Table 2).

#### 3.4.2. Time-Series Studies of Outdoor Air Pollution Studies

The study quality of the only time-series study we selected was 19 out of 23 (Table S4). In a hospital based time-series analysis, performed on an hourly basis, Gestro et al. reported that higher ambient NO_2_ concentrations were associated with a higher risk for OM hospital admissions, with a 0–8 h lag (RR 1.03; 95% CI: 1.01–1.05), and subsequent ones up to lag range 0–15 h [40] (Table 2).

### 3.5. Studies Investigating cross Sectional Associations between Air Pollution and OM

#### 3.5.1. Case Control Studies of Indoor Air Pollution

The quality of the two case control studies was good (NOS = 5). Neither were adjusted for important confounders and this may have overestimated the reported strength of associations (Table S1) [30,31].

The two case control studies that we selected, investigated the effects of indoor air pollution on OM in children [30,31]. Daigler et al. [30] reported a statistically significant positive association between current wood burning stove use in U.S. households and higher odds for OM in children (OR 1.73; 95% CI: 1.03, 2.89), using 125 cases and 246 controls [30]. Da Costa et al. [31] investigated effects of indoor smoke exposure in children younger than 6 years in one region of Mozambique. Cases were defined as children having type B tympanograms in one or both ears, and classified as having middle ear effusion (*n* = 307). Controls were recruited from the same village or neighbourhood as the cases, and matched by age (±4 months) and sex (*n* = 443). They reported strong associations between charcoal or wood smoke exposure and OM in children aged <2 years (OR 3.09; 95% CI: 2.00, 4.78) and aged >2 years (OR 3.18; 95% CI: 2.01, 5.01) [31]. Interestingly, the effect estimates for indoor tobacco smoke exposure and OM appeared to be weaker than for wood or charcoal smoke exposure, among children <2 years (OR 2.81; 95% CI: 1.64, 4.80) and >2 years (OR 1.61; 95% CI: 1.13, 2.30) (Table 3).

#### 3.5.2. Cross Sectional Studies of Outdoor Air Pollution Exposure

The overall quality of the cross sectional studies ranged from low to medium (score range from 3 to 6 (Table S5). Of eight cross sectional studies that we selected, five investigated OM in children with specific outdoor air pollutant concentrations obtained from fixed site exposure monitoring stations; none used modelled air pollution exposure levels [32,35,37,38,39]. A single study compared OM in rural and urban areas [34]. Two other studies compared OM in children who lived closer and farther away from industrial sources [33,36]. Bhattacharyya et al. analysed aggregated cross-sectional US National Health Interview Survey data coupled to U.S. EPA data reported annually, from 1997 to 2006 [32]. The authors found that lower criteria pollutant concentrations (i.e., NO_2_, SO_2_, CO and PM) were associated with a lower prevalence of frequent childhood OM, defined as ≥3 ear infections in the previous 12 months [32]. Similarly, Heinrich et al. [38] reported a lower childhood OM prevalence with lower SO_2_ and total suspended particles (TSP) concentrations in Germany. A small cross sectional study of 323 school children from Sao Paulo, Brazil aged 11–13 years reported that residence in high air pollution areas, defined based on mean levels of SO_2_ and PM, had a higher prevalence of ear infections compared to low pollution areas [39]. However, another small cross sectional study, from Croatia, found no evidence of correlations between levels of SO_2_ or smoke and surgically confirmed SOM [37]. Two additional cross sectional studies reported statistical evidence that closer residence to industrial air pollution sources was associated with a higher prevalence of OM in children [33,36]. One study reported a statistically significant trend for lower “Glue Ear” prevalence among children residing at increasing distances from a British coking works, relative to children living closer [33]. The other study, also from the UK, found a statistically significant higher proportion of children with OME living within 1000 m of an industrial air pollution source compared with those who lived farther way [36]. Finally, Harvey et al. [34] reported that urban children had a higher incidence of OM compared to rural children (Table 3).

## 4. Discussion

Since 2004, there has been increasing evidence for evidence of an association between ambient air pollution exposure and a higher risk of OM in children. Prospective birth cohort studies provide the strongest available evidence to date. However, there are inconsistencies in the results between studies, which may be related to methodological differences in exposure assessment strategies, the types of air pollutants examined (e.g., traffic, PAHs, benzene, wood smoke, etc.) and the timing of exposure windows (prenatal and post-natal), as well as geographical variations. Despite this, all studies investigating this association found evidence that exposure to at least one pollutant increased the risk of OM in young children. In birth cohort studies that considered exposure to a range of pollutants, NO_2_ showed the most consistent positive association while the other pollutants showed no associations. In the largest birth cohort study that combined 10 European birth cohorts, a significant positive association between NO_2_ and OM was reported [13]. The case-crossover and time-series studies also found significant associations between outdoor air pollution exposure and ED attendances for OM. Both case control studies from the USA and Mozambique investigated indoor air pollution from heating and cooking, especially related to wood smoke exposure, and found significant associations with OM [30,31]. 

### 4.1. Outdoor Air Pollution Exposure and OM

A diverse array of air pollutant exposures was investigated in studies related to outdoor air pollution that we reviewed here. The majority of studies measured U.S. National Ambient Qir Quality Standard criteria pollutants: PM_2.5_, PM_10_, SO_2_ and NO_2_. Overall, there is persuasive evidence for air pollutants increasing the risk of OM in children, but there is some heterogeneity. Findings from the birth cohort and time-series studies indicated evidence for a link between NO_2_ exposure and OM in children [13,23,24,44], but some studies reported no association [13,29]. Similarly, the associations between particulate matter exposure and OM were not consistent between studies. There was a trend towards positive associations in the birth cohort studies but the effect estimates were not statistically significant [13,14,24,29]. Retrospective cohort studies from China found positive associations between SO_2_ exposure and OM in children [29] but some cross-sectional studies reported only a trend [32,38,39]. This heterogeneity may be partly related to different methodologies to assess exposure [29]. For example, MacIntyre et al. 2011 used two methods to assign outdoor air pollution, (1) a LUR model and (2) Inverse-Distance-Weighted concentrations (IDW). The association between PM_2.5_ and OM was not evident when using the LUR classification of exposure but was found for exposure measured by IDW [28]. A single study investigated the link between PAH exposure and OM in children. The Spanish INMA birth cohort modelled outdoor benzene and found that maternal exposure during the first two trimesters was associated with OM in children during the first 12–18 months of life [23]. Immunotoxic properties of PAHs may impair the immune function of the fetus, and subsequently increase susceptibility to OM during early life.

### 4.2. Indoor Air Pollution Exposure and OM

Biomass burning is a major source of air pollution in most of the developing countries, and one of the major contributors to indoor air pollution, as it is an important heating and cooking fuel in low income countries. Not only in low income countries, wood heaters or fire places are also common in some developed countries. The findings from studies of wood smoke exposure and increased risk of OM in children are consistent with toxicological effects of wood smoke on lung epithelial cells [28,30,31]. Constituents of wood smoke increase epithelial cell oxidative stress in experimental studies [46]. A recent experimental study, demonstrated cytotoxic and genotoxic effects in human alveolar and bronchial cells exposed to particulate matter from Beech wood chips smoke, that was comparable to that caused by diesel PM [47]. A Canadian study argued that wood smoke effects might be caused by cytotoxicity, or simply be an artefact of the strong seasonal correlation between wood burning and OM in children [28]. A case control study from Mozambique demonstrated a strong link between household charcoal and/or wood and OM [31]. Especially in low income countries exposure to indoor biomass smoke is higher for pregnant women and young children since women do the majority of cooking and their children accompany them during these times. New information from two Chinese studies provided evidence that indoor air pollution from house renovation and new furniture were associated with OM in children [29]. 

The Polish birth cohort study used personal monitors to detect maternal exposure to PAHs during pregnancy, and found an elevated risk of increased number and intensity of OM in children from birth to 1 year of age [26]. This study highlights the importance of PAHs in the indoor air pollutant mixture, and how prenatal exposure to these agents may be related to infant OM.

### 4.3. Evidence from Long-Term, Lag and Cross Sectional Studies

Long-term studies investigating effects of outdoor air pollution exposure and OM in children showed an association between NO_2_ exposure and increased risk. Other pollutants, however, showed inconsistent associations. Case crossover and time series studies investigating lag effects of air pollution on OM did not find conclusive evidence. Two case control studies that measured indoor air pollution and OM cross sectionally found consistent increased risks. Although these studies did not account for long term exposures, current indoor air pollution exposure frequently represents longer exposure periods. However, the results should be interpreted with caution, as neither study adjusted for confounding [30,31]. Cross sectional studies mainly investigated the difference of OM prevalences between high and low polluted areas, and found higher prevalence in high polluted areas compared to low polluted areas.

Overall, the findings from these studies suggest that both outdoor and indoor sources of air pollution are associated with OM in young children. This is biologically plausible in light of recent publications concerning the pathological processes that can lead to OM [16,48]. ETS exposure was not considered in this review, however, a recent systematic review and meta-analyses suggested a strong link also between ETS exposure and OM in children. In this review living with a smoker was associated with a 62% increased risk of middle ear diseases in children [10].

### 4.4. Biologic Mechanisms

OM is commonly caused by viruses and bacteria migrating from the nasopharynx, through the Eustachian tube into the middle ear. Obstruction of the Eustachian tube leads to accumulation of mucus in the middle ear and an ideal environment for pathogen growth. If pathogens are also present this can lead to OM. Recent experimental studies shed some light on the mechanisms and pathophysiology associated with air pollution exposure and OM. These studies investigated effects of air pollution in terms of cytotoxicity, inflammation and increased expression of mucin. Further, studies used microarray and pathway analyses to identify gene expressions and molecular pathways related to particulate matter exposure and OM.

In an in vivo mouse study, a transtympanic injection of urban particulate matter (UPM), administered into the middle ear cavity, induced histological mucosa changes characteristic of inflammatory responses [49]. In an in vitro study of the effects of vehicle emissions on the human ear, immortalized human middle ear epithelial cell lines (HMEECs) were exposed to diesel exhaust particles (DEPs) [48,49]. The authors reported that after DEP exposed the HMEECs had decreased cell viability, as well as increased expression of mucin and inflammatory cytokines [48,49]. The researchers also reported increased expression of 5AC, oligomeric mucus/gel-forming (MUC5AC) gene, upon DEP exposure [48,49]. MUC5AC is exclusively produced by mucus-secreting epithelial goblet cells, and has been identified as a major contributor to the rheological properties of respiratory tract epithelium mucus, and has plays a vital role against pathogenic and environmental challenges [50,51]. Increased MUC5AC expression has been also detected in HMEECs exposed to tobacco smoke and in mouse middle ear injected with UPM [49,52]. The same study reported increased HMEEC expression of inflammatory cytokines, such as tumor necrosis factor-α (TNF-α) and cyclooxygenase-2 (COX-2), following DEP exposure [48].

Song et al. demonstrated that exposure to particulate matter caused changes in the regulation of 611 genes in HMEECs, of which 366 were up-regulated and 245 were down-regulated [16]. The up-regulated genes were mainly involved in generation of reactive oxygen species, inflammatory response, immune response, cell proliferation, apoptosis and cell differentiation. The authors identified 21 genes as important components of a potential signaling network and 25 genes as modulators in the signaling pathway. They have identified four genes: Vascular endothelial growth factor (VEGFA), Interleukin 1 beta (IL1B), Granulocyte-macrophage colony-stimulating factor (CSF2) and Heme oxygenase (decycling) 1 (HMOX1) as mediator genes of the up-regulated genes; and another four genes: Insulin-like growth factor 1 receptor (IGF1R), Tissue inhibitor of metalloproteinase 1 (TIMP1), Interleukin 6 (IL6) and Fibronectin 1 (FN1), as modulators among the down-regulated genes [16]. 

Kim et al. performed an in vivo study using mouse models to identify biomarkers in the middle ear related to DEP exposure [15]. They identified altered expression of numerous genes after exposure to DEP that related to the immune response in diverse cellular processes. Most of the expressed genes were related to Interleukin 2 (IL2) expression and T-cell maturation. Of the down-regulated genes, Cluster of differentiation 4 (CD4), Interferon Alpha 1 (IFNα1) and Estrogen Receptor 1 (ESR1) were related to immune regulation, and Son of Sevenless homolog 1 (SOS1) known to be linked with several signaling pathways related to T-cell responses. Up-regulation of coactivator-associated arginine methyltransferase 1 (CHRM1) and erythropoietin (EPO) in mice middle ear epithelium after DEP exposure links with epithelial cell proliferation, erythropoiesis and production of red blood cells [15].

These in vivo and in vitro studies provide evidence that particulate matter related to vehicle emissions has effects on the middle ear through inducing inflammatory responses and increasing mucin production. However, there is a limited understanding of the mechanisms behind air pollution exposure and OM. Although it is plausible to postulate that these increases in mucin and inflammation would lead to Eustachian tube blockage and any pathogens finding their way into this ideal environment for colonization and reproduction may cause infection

### 4.5. Strengths and Limitations

A major strength of this review is that inclusion of all the published epidemiological studies investigating ambient air pollution and OM in children. We investigated the reviewed studies for publication bias with a funnel plot, using NO2 exposure during birth or the first year of life and subsequent OM in children. NO2 exposure is the most commonly reported exposure and outcome relationship. The funnel plot did not indicate strong evidence for publication bias (Figure S1). We have included a range of observational study designs in this systematic review, from birth cohorts to cross sectional studies, in order to synthesise most of the available evidence. The validity and reliability of evidence generated differed by study design, with birth cohort studies having provide the highest level of evidence and cross sectional studies the lowest due to basic methodological biases inherent in study designs. Key methodological issues in the studies included in this review were exposure assessment and how OM was defined. Most birth cohort studies used advanced land use regression models to assign early life point location exposure based on residential location, with modest potential for misclassification. However, most of the other studies used either fixed site monitoring stations or dichotomous urban/rural classification, which raise the issue of potential exposure misclassification. In either case, exposure misclassification is unlikely to have been differential among those with and without OM and so reported associations may have underestimated the true, underlying population effect. There are strengths and limitations in different types of air pollution assessment methods used in epidemiological studies. Fixed site air quality monitoring stations provide highly resolved temporal variations of air pollution data but they do not provide spatial variation of air pollutants. Proximity variables, for example “distance to roads” do not consider wind directions, traffic volume, and composition. LUR methods compute individual air pollution exposure considering a number of parameters such as traffic, population density, land use, and vegetation. Although the LUR approach is considered superior to other methods, its validity depends on the tenability of the model assumptions [53]. Case definitions for OM varied substantially between studies we reviewed. Some studies used parental report of a doctor-diagnosed OM which may be subject to recall bias and therefore is less reliable than a diagnosis documented by a health professional. In particular, it may be unclear whether this related to the acute infection, its complication, or possibly another upper respiratory tract pathology. Furthermore, case control designs relying on parental questionnaire might introduce a recall bias, in which case parents report more accurate exposure histories than controls, potentially leading to an overestimated effect. In addition, heterogeneity in the type of OM may have obfuscated diagnosis-specific association with air pollution exposure, as some studies allowing for an array of diseases with differing pathophysiologies, including AOM, OME and CSOM, and some studies including only one type of OM (most commonly AOM).

Most studies we reviewed, especially prospective birth cohort studies, were from Europe or North America. There were only two studies from developing countries: a single cohort from China [29] and a case control study from Mozambique [31]. Given that low-income countries bear the largest burden of disease in terms of sequelae and deaths from OM as well as highest levels of air pollution [6], the effects of outdoor and indoor air pollution on OM in these countries may be much higher. We only included articles in English, and this may have contributed to some selection bias. Due to the heterogeneity of included studies in this systematic review, we did not perform a meta-analysis. The findings from the ESCAPE Project [13] meta-analysed 10 European birth cohorts and some of these cohorts have published their findings individually. The case control, case-crossover, and time-series studies did not report the same exposure and outcome in at least two studies and were heterogeneous in exposure definitions.

## 5. Conclusions

This systematic review found that the evidence for a causal link between exposure to air pollution and OM in infants and children is strengthening, yet remains limited. Although there was a general consensus that higher levels of exposure to NO_2_, PM, SO_2_, PAH and wood smoke levels are associated with a prevalence of infections of OM the effect estimates varied. Importantly, we did not identify any birth cohort studies conducted in low income countries, where high air pollution levels occur and the communities bear highest a high burden of OM. Although recent experimental studies shed some light into the biomarkers and mechanisms of air pollution exposure and OM, a clear mechanism has not yet been identified.

## Figures and Tables

**Table 1:** Summary of air pollution exposure and otitis media in long-term studies.

Cohort Studies	Study, Country and Sample	Study Design	Exposure Assessment & Age	Outcome Assessment & Age	Time between Exposure and Outcome	Results				Adjustment for Confounders
	Aguilera [23] INMA study, Spain—2199 infants	General population birth cohort study, recruited 2003–2008	NO_2_ and benzene assigned using temporally adjusted LUR during pregnancy and 1st year of life. For those who changed residential address an average weighted exposure was determined	Parent reported physician diagnosed ear infections (AOM) during the first 12–18 months of life. (one or more—not differentiated)	Cumulative exposure 12–27 months	Exposure to ambient air pollution and risk of OM				Birth season, child care attendance, siblings at birth, parental asthma and allergy, BMI-pre-pregnancy, ETS exposure during pregnancy, and postnatal parental smoking
						Exposure period	RR 95% CI NO_2_ (per 10 μgm^−3^)		RR 95% CI Benzene (per 1 μgm^−3^)	
						Entire prenatal	1.18 (0.98, 1.41)		1.13 (0.95, 1.34)	
						1st trimester	1.11 (0.99, 1.24)		1.08 (1.02, 1.15)	
						2nd trimester	1.16 (0.98, 1.37)		1.13 (1.00, 1.27)	
						3rd trimester	1.12 (0.98, 1.29)		1.02 (0.92, 1.13)	
						1st year of life	1.15 (1.01, 1.31)		1.08 (0.99, 1.18)	

	Brauer [24] PIAMA study, Netherlands —2984 infants (1st year) —2970 infants (2nd year) LISA study, Germany —620 infants (1st year) —605 infants (2nd year)	General population birth cohort studies, PIAMA: recruited 1997–1998 LISA, recruited: December 1997–January 1999	NO_2_, PM_2.5_ and black carbon levels at the birth address using LUR. Long term average concentrations were calculated for home address at time of birth	Parent reported physician diagnosed infectious OM during the first 2 years of life (any episode)	2 years	Exposure to ambient air pollution and odds of OM				Maternal smoking during pregnancy, ETS exposure, maternal/paternal education, sex, gas use for cooking/heating, siblings, breastfeeding, moulds, pets, parental allergy, maternal age. In the Netherlands only, adjusted for ethnicity, study arm (intervention/natural history), and use of allergen-impermeable mattress cover.
						Exposure period & pollutant (per µg/m^−3^)	OR (95% CI) Netherlands		OR (95% CI) Germany	
						1st year of life	*n* = 2984		*n* = 620	
						PM_2.5_ (10)	1.13 (0.98, 1.32)		1.19 (0.73, 1.92)	
						Black carbon (0.5)	1.11 (0.98, 1.26)		1.12 (0.83, 1.51)	
						NO_2_ (3)	1.17 (1.03, 1.34)		1.09 (0.78, 1.54)	
						Cumulative 2nd year of life	*n* = 2970		*n* = 605	
						PM_2.5_ (10)	1.13 (1.00, 1.27)		1.24 (0.84, 1.83)	
						Black carbon (0.5)	1.10 (1.00, 1.22)		1.10 (0.86, 1.41)	
						NO_2_ (3)	1.14 (1.03, 1.27)		1.14 (0.87, 1.49)	

	Deng [29] Children attending kindergartens in Changsha, Hunan Province in south-central, China—1617 children	Retrospective cohort study, 2011–2012	NO_2_, PM_10_ and SO_2_ measured using fixed site monitors. Exposure to indoor air Pollution related to home renovation activities were surveyed by a questionnaire. Air pollution during prenatal and (postnatal) first year to the past year	Parent reported physician diagnosed life-time prevalence of infectious OM, reported at age 3–4 years	Cumulative exposure 3–4 years	Exposure to air pollution and OM				Child’s sex, birth season, breastfeeding, day-care-attendance age, parental atopy, parental SES by house size, ETS at home, visible mould/damp stains, window condensation, dogs in house, and cockroaches in house
						Single Pollutant (per µgm^−3^)	OR (95% CI) Prenatal		OR (95% CI) Postnatal & past year	
						PM_10_ (prenatal: 7 µgm^−3^; postnatal: 6 µgm^−3^)	0.95 (0.73, 1.23)		1.36 (0.95, 1.94)	
						SO_2_ (prenatal: 27 µgm^−3^; postnatal: 13 µgm^−3^)	1.44 (1.09, 1.88)		1.32 (0.95, 1.84)	
						NO_2_ (prenatal: 12 µgm^−3^; postnatal: 13 µgm^−3^)	1.10 (0.76, 1.60)		1.16 (0.73, 1.83)	
						Postnatal exposure to new furniture and redecoration were significantly associated with OM respectively OR 1.62 (95% CI 1.05, 2.49) and OR 1.81 (95% CI 1.12, 2.91). Prenatal exposure to outdoor SO_2_ and postnatal exposure to indoor renovations was associated only with 1–2 episodes of OM [SO_2_ OR = 1.46 (95% CI 1.09, 1.95); new furniture OR = 1.76 (95% CI 1.14, 2.72); Redecoration OR = 1.93 (95% CI 1.18, 3.16)] but not with ≥3 episodes.				
	Deng [45] Children attending kindergartens in Changsha, Hunan Province in south-central, China—1617 children	Retrospective cohort study, 2011–2012	NO_2_, PM_10_ and SO_2_ measured using fixed site monitors. Exposure to indoor air pollution related to home renovation activities were surveyed by a questionnaire. Air pollution exposure during 1st, 2nd and 3rd trimester	Parent reported physician diagnosed life-time prevalence of infectious OM reported at age 3–4 years	Cumulative exposure 3–4 years	Exposure to air pollution and otitis media				Child’s sex, birth season, breastfeeding, antibiotic use, parental atopy, parental SES by house size, ETS, new furniture, house redecoration, visible mould/damp stains, window condensation, household pets, and cockroaches in house
						Single Pollutant (per µgm^−3^)	OR (95% CI) 1st trimester	OR (95% CI) 2nd trimester	OR (95% CI) 3rd trimester	
						PM_10_ (15, 14, 16, respectively)	0.91 (0.67, 1.26)	1.03 (0.77, 1.39)	0.89 (0.65, 1.22)	
						SO_2_ (42, 32, 38, respectively)	1.46 (1.04, 2.03)	1.40 (1.06, 1.84)	1.44 (1.02, 2.03)	
						NO_2_ (17, 15, 14, respectively)	0.89 (0.57, 1.37)	1.20 (0.83, 1.74)	1.10 (0.77, 1.56)	
						Prenatal SO_2_ exposure was not associated with the repeated attacks, but with onset of OM, OR 1.47 (1.10, 1.96); association was stronger in females and children with parental atopy; children living in houses with window condensation and noticeable cockroaches				
	Dostal [25] Teplice and Prachatice, Czech Republic—960 children	Birth cohort followed up to 10 years, Recruited 1994–1999 (NB: only first 2 years’ results were reported)	Compared participants living in more polluted industrial district of Teplice with those in the less polluted rural district of Prachatice Exposure data—town of birth	Used ICD-10 codes H65–67 and H92 which covers serous and infectious OM along with complications like perforation and mastoiditis	2 years	In the first two years, the children living in the urban and industrial environment of Teplice district had a significantly higher incidence of OM compared to children living in the rural district of Prachatice, Rate ratio 2.3 (95% CI 1.7–4.1). Beyond 2 years of age the differences were not significant.				Gender, ethnicity, maternal age and education, the two parts of the study (at 3 and 4.5 years), season of birth, maternal history of allergy, preterm birth and/or low birth weight, and atopic dermatitis of children
	Jedrychowski [26] Krakow, Poland —333 infants	Birth cohort up to 1 year. Recruited November 2000–August 2002	Polycyclic aromatic hydrocarbons (PAHs) assigned using prenatal personal air monitoring of mothers in the second trimester of pregnancy over a 48-h period	Parent reported ear infections (OM). Number of episodes and duration reported during 3 months in the first year of life.	6 months	Increased risk for prenatal PAH exposure and number and duration of ear infections during the first year of life, per log unit of PAH concentration (ngm^−3^) RR 1.82 (95% CI 1.03, 3.23) and RR 1.64 (95% CI 1.34–2.00), respectively Prenatal PAH exposure and number and duration of ear infections during the first year of life (dichotomized by median values of PAH distribution) RR 2.00 (95% CI 1.09–3.65) and RR 2.05 (95% CI 1.66–2.52), respectively				Increased risk for prenatal PAH exposure and number and duration of ear infections during the first year of life, per log unit of PAH concentration (ngm^−3^) RR 1.82 (95% CI 1.03, 3.23) and RR 1.64 (95% CI 1.34–2.00), respectively Prenatal PAH exposure and number and duration of ear infections during the first year of life (dichotomized by median values of PAH distribution) RR 2.00 (95% CI 1.09–3.65) and RR 2.05 (95% CI 1.66–2.52), respectively				Child’s sex, birth weight, season of birth, ETS in postnatal period, mother’s allergy, mother’s education level, moulds at home
	MacIntyre [13] 10 European birth cohorts within the ESCAPE Project: BAMSE (Sweden), GASPII (Italy), GINIplus and LISAplus (Germany), MAAS (United Kingdom), PIAMA (Netherlands), and four INMA cohorts (Spain), —16,059 children	Follow up from birth to 2 years (6 months–3 years depending on cohort)	Assigned NO_2_, NO_X_, PM_2.5_, PM_2.5_ absorbance, PM_10_, and PM_2.5–10_ exposure at birth using LUR models	Parent report of physician-diagnosed OM (not specified)	6 months–3 years depending on included cohort	Combined results for air pollution exposure during birth and OM from birth to 2nd year of life				Municipality (BAMSE), sex, older siblings, breastfeeding at 6 months, parental atopy, child-care, maternal smoking during pregnancy, any ETS in the child’s home, visible mould or dampness in the home, use of gas stove, birth season, parental SES, and intervention (GINIplus, MAAS, PIAMA).
						Pollutant		OR (95% CI)		
						NO_2_ (per 10 µgm^−3^)		1.09 (1.02, 1.16)		
						NO_X_ (per 5 μgm^−3^)		1.05 (0.98, 1.12)		
						PM_2.5_ (per 5 µgm^−3^)		1.06 (0.75, 1.49)		
						PM_2.5_ absorbance (per 1 unit)		1.08 (0.83, 1.39)		
						PM_2.5–10_ (per 5 µgm^−3^)		0.97 (0.88, 1.08)		
						PM_10_ (per 10 µgm^−3^)		0.98 (0.84, 1.14)		

	MacIntyre [28] South-west British Columbia, Canada—45,513 infants (a retrospective cohort)	Follow up from birth to 2 years. 1999–2000	(1) Inverse-distance weighting of monitor data (CO, NO, NO_2_, O_3_, PM_2.5_, PM_10_, SO_2_); (2) LUR model (NO, NO_2_, PM_2.5_, black carbon, wood smoke); (3) proximity to roads and point sources. Estimated 24 months of life	Outpatient physician visits codes for infectious and serous OM from a series of linked administrative datasets obtained from the BC Ministries of Health, Vital Statistics Agency, and Perinatal Database Registry.	Cumulative exposure 24 months	Air pollution exposure during birth and OM from birth to 2nd year of life (pollutants from LUR)				* sex, Aboriginality, older siblings, maternal smoking during pregnancy, maternal age, neighbourhood income, neighbourhood female education.
							Adjusted for *		Adjusted for * + OM season	
						Pollutant	OR (95% CI)		OR (95% CI)	
						NO_2_ (per 10 µg/m^−3^)	1.09 (1.07–1.12)		0.97 (0.95, 0.99)	
						NO (per 24.1 µgm^−3^)	1.18 (1.16–1.21)		1.10 (1.07, 1.12)	
						PM_2.5_ (per 1.8 µgm^−3^)	0.91 (0.89–0.93)		0.99 (0.97, 1.01)	
						Black carbon (per 1.1 µgm^−3^)	0.94 (0.93–0.96)		0.99 (0.97, 1.01)	
						Wood smoke (per 16 days)	1.51 (1.47–1.55)		1.32 (1.27, 1.36)	

	Pettigrew [27] Virginia, U.S.—904 infants	Birth cohort study, 3 months to 1 year follow up. Participants were selected based on use of regular kerosene heater or gas stove. Non-smoking mother and households. 1993–1996	Self-reported number of hours each secondary heating source was used in the home during that reporting period	Mother reported physician diagnosed ear infections during telephone interviews every 14–19 days for the 1st year of life.	9 months	Association of indoor secondary heating sources and OM				Heating season, gas appliances in the home, infant sex, season of birth, race, mother’s education, other children in the household, duration of breastfeeding, mother’s allergies, mother’s asthma, reported mold in the home, pet cat or dog
							Average daily use—unadjusted		Average daily use—adjusted	
						Type of heating—per 8-h/day increase	OR (95% CI)		OR (95% CI)	
						Fireplace	1.51 (1.17–1.93)		1.25 (0.92–1.69)	
						Wood stove	1.14 (1.00–1.29)		1.01 (0.52–1.23)	
						Kerosene heater	1.05 (0.92–1.21)		1.07 (0.92–1.26)	


**Table 2:** Summary of air pollution exposure and otitis media in studies investigating lag effects.

Case Crossover studies	Study, Country and Sample	Study Design	Exposure Assessment & Age	Outcome Assessment & Age	Time between Exposure and Outcome	Results				Adjustment for Confounders
	Strickland, Hao [42] Georgia, USAChildren aged 0–18 years attending ED (*n* = 8,252,559)	Time-stratified, ecological case crossover 1 January 2002 to 30 June 2010	Aerosol Optical Depth derived from MODIS satellite	ED visits to 150 hospitals extracted from Georgia Hospital Association. OM was identified by discharge diagnosis of OM using ICD—9 codes (381 or 382)—both non-suppurative and suppurative and not specified OM	Air pollution and OM measured at same time (cross-sectionally lag 1)	Odds ratios per 10-μgm^−3^ increase in same-day PM_2.5_ and OM ED visits Lag 0 OR = 1.005 (95% CI 0.996, 1.014) Lag 1 OR = 0.995 (95% CI 0.985, 1.004)				Temperature, mean humidity, day of year, day of week, warm season, holiday, lag holiday
	Xiao, Liu [43] Georgia, USA Children aged 0–18 years attending ED (*n* = 422,268)	Time-stratified, ecological case crossover 1 January 2002 to 31 December 2008	Daily pollutant concentration at 12 km spatial resolution, estimated from CMAQ model simulations: CO, NO_2_, SO_2_, O_3_, PM_10_, PM_2.5_, SO_4_^=^, NO_3_^−^, NH_4_^+^, EC, OC	ED visits to 150 hospitals for OM extracted from Georgia Hospital Association. OM was identified by discharge diagnosis of OM using ICD-9 codes (381 or 382).	Up to 3 day 3-day lag between air pollution measurement and ED presentations for OM	ORs for interquartile range increases in 3-day moving average ambient air pollutant concentrations. Traffic pollutants (CO, NO_2_, EC, and OC) associated with increased risk of OM Joint effects (with interactions) OR = 1.025 (95% CI 1.012, 1.039) Joint effects (without interactions) OR = 1.018 (95% CI 1.010, 1.026)				Temperature, humidity, warm season, holiday, lag holiday
	Zemek [44] Canada—14,527 children (aged 1–3 years, over 10 year period) who attended ED for OM	Time-stratified ecological case crossover study	Data for CO, NO_2_, O_3_, SO_2_, and PM_10_ and PM_2.5_ were obtained from fixed site monitoring stations.	OM was identified by discharge diagnosis of OM using ICD-9 rubric (code 382.9 only)	Up to 4-day lag between air pollution measurement and ED presentations for OM	Associations between IQR increase in pollutants and OM ED visits based on lag times (days)				Temperature, and relative humidity
						Pollutan	Lag	Warm months OR (95% CI)	Cold months OR (95% CI)	
						CO	1	1.08 (1.00, 1.17)	0.98 (0.96, 1.00)	
							2	1.14 (1.06, 1.23)	1.02 (0.99, 1.04)	
							3	1.08 (1.00, 1.16)	1.00 (0.98, 1.02)	
						NO_2_	2	1.10 (1.02, 1.19)	1.03 (1.00, 1.07)	
							3	1.08 (1.00, 1.17)	0.99 (0.96, 1.03)	
						O_3_	1	1.01 (0.93, 1.09)	1.07 (1.01, 1.14)	
						PM_10_	2	1.05 (1.00, 1.10)	1.01 (0.97, 1.05)	
							4	1.05 (1.00, 1.10)	1.00 (0.96, 1.04)	
						Overall the strongest associations were during the warm months compared to cold months				
	Kousha [41] Ontario, Canada —4815 children aged ≤3 years	Time-stratified, ecological case crossover study of ED visits for OM	Air Quality Health Index (AQHI)	ED visits with ICD-10 codes H65 (nonsuppurative OM) and H66 (suppurative and unspecified OM) used to identify visits for conditions related to acute and chronic middle ear inflammation.	Up to 15-day lag between air pollution measurement and ED presentations for OM	Odds ratio for 16.5 ppb (IQR) increase in O_3_ and 8.2 µgm^−3^ (IQR) increase in PM_2.5_ and OM ED visits				Humidity, temperature
						Pollutant	Lag days	OR (95% CI)		
						O_3_ (8 h)	6	1.16 (1.02, 1.31)		
							7	1.20 (1.05, 1.34)		
						PM_2.5_	3	1.07 (1.01,1.13)		
							4	1.07 (1.01, 1.13)		
						For every 1 unit increase in the AQHI, discharge diagnosis of OM increased 5% to 6% three days post exposure Distributed Lag Nonlinear Model ResultsThe overall risk for OM, in the first 15 days after an increase in the AQHI, was 1.22 times the risk of OM on days following no increase in exposures				
**Time-Series**	Gestro [40] Cuneo, Italy —2532 children and adolescents aged 0–18 years attending ED with OM.	Hospital based time series analysis 2007–2010	PM_10_, NO_2_, O_3_, CO from a fixed site monitoring station	Diagnoses were coded according to the ICD-9 coding for OM (1st and 2nd diagnosis)		Modest association between increased NO_2_ and attending ED with OM, RR 1.03 (95% CI 1.01,1.05) with a lag time range from 0–8 h per 10 μgm^−3^ of NO_2_				Upper respiratory tract infections, influenza, seasonality

**Table 3:** Summary of air pollution exposure and otitis media in studies investigating exposure and outcome cross sectionally.

Case Control Studies	Study, Country and Sample	Study Design	Exposure Assessment	Outcome Assessment	Results					Adjustment for Confounders
	Daigler [30], USA —371 children attending 4 private paediatricians	Case-control study 1986–1987	Parental questionnaire about housing conditions, type of heating fuel used and type of cooking stove used.	Cases defined as children with ≥2 visits for separate OM episodes diagnosed using pneumatoscopy. Controls were children seen for routine health maintenance without acute diseases.	Wood burning stove associated with OM children OR 1.73 (95% CI 1.03–2.89)					None
	da Costa [31], Maputo, Mozambique —750 children <6 years old from a hospital and its catchment area.	Case-control study	Research nurse administered questionnaire to parents: exposure to wood smoke, charcoal smoke and other covariates.	Cases were the children having type B tympanograms in one or both ears, and classified as having middle ear effusion. Controls were recruited from the same village or neighbourhood as the cases, and matched by age (±4 months) and sex.	Charcoal or wood use in home associated with OM in children <2 years of age OR 3.09 (95% CI 2.0, 4.78). Charcoal or wood use in home associated with OM in children >2 years of age OR 3.18 (95% CI 2.01, 5.01).					Sex and age
**Cross sectional analysis**	Harvey [34], UK —4949 school children aged 8–16 years	Cross-sectional analysis of a cohort during 8 years. 1964–1971	Living in rural or urban area	Secretory OM diagnosed at a clinic—“cases where aspiration under general anaesthetic resulted in a fluid ranging from a thin, easily-evacuated fluid to the “glue” of the so-called glue ear.”	Incidence of secretory OM = 13.1 per 1000 school children in the rural area; and 19.8 per 1000 school children in the city area. No statistical tests were reported					None
	Bhattacharyya [32] The National Health Interview Survey (USA) —126,060 children	Cross sectional samples from 1997–2006	CO, NO_2_, SO_2_ and PM data from fixed site monitors	Parent reported 12-month prevalence of ≥3 ear infection episodes	Lower prevalence of ear infection was found to be related to the lower levels of ambient criteria pollutants (i.e., improvements in air quality). Regression coefficient *p*-value < 0.001 for all pollutants.					None
	Bhopal [33] UK—“green field” coking works sites —685 children (ages not reported)	Cross sectional—participants living closer and farther away from a coking works site, during 1990–1991	Exposure to coking works categorized into inner, outer and control groups.	Prevalence of “Glue Ear” was collected using a postal survey	Living closer to coking works and reported “Glue Ear” in children 0–15 years					None
						Inner (%)	Outer (%)	Control (%)	Chi^2^ trend p	
					“Glue Ear”	15.2	8.6	9.0	<0.02	
	Heinrich [38] former East Germany —4949 children aged 5–14 years	Three repeated cross sectional surveys: 1992–1993, 1995–1996, and 1998–1999	SO_2_ and Total Suspended Particles (TSP) measured using a mobile and fixed monitoring stations	Parent reported child ever diagnosed with OM by a physician	Increasing air pollution exposure and OM in children					Age, sex, parental education, parental atopy, home dampness or moulds, gas cooking, ETS at home and contact with cats
						TSP (50 µgm^−3^ Increment) OR (95% CI)		SO_2_ (100 µgm^−3^ Increment) OR (95% CI)		
					Prevalence of OM	1.45 (0.89, 2.37)		1.42 (0.94, 2.15)		

	Heinrich [35] former East Germany —3785 children aged 5–14 years	Two repeated cross sectional surveys: 1992–1993 and 1995–1996	Air pollution measured using fixed monitoring stations and specific monitors placed for the study	Parent reported child ever diagnosed with OM by a physician	Decreased prevalence of OM (30.9% versus 26.3%) was found between the 1992–1993 survey and the 1995–1996 surveys Decreased prevalence of OM was observed between the two surveys OR: 0.83; (95% CI: 0.73 to 0.96). Associations with changing levels of air pollutants not reported					Area, age, parent education, birth weight, breastfeeding, parental atopy, house status, house living space, bedroom share, dampness, type of heating, presence of carpet, ETS exposure, mother’s smoking during pregnancy, contact with cats and day care attendance
	Holtby [36], UK —1116 School entrant children	Cross sectional analysis 1988–1992	Distance between children’s home address and industrial emission source	OME defined by clinical examination using pure tone audiometry and auto admittance	Statistically significant higher proportion of children with OME lived within a 1000-m buffer from an industrial pollution source than >1000-m from an industrial pollution source.					None
	Ribeiro [39] Sao Paulo, Brazil —323 children aged 11–13 years, living in 3 areas with different air pollution levels: low, intermediate and very high.	Cross sectional analysis 1998	Air pollution data obtained from fixed site monitoring stations, mainly for SO_2_ and PM	Parent reported ear infections	Higher prevalence of ear infections in the more polluted areas than in the less polluted areas					None
	Sprem [37], Zagreb, Croatia —297 children aged 1–10 years admitted to one hospital for Secretory OM (SOM)	Hospital based cross sectional study 1981–1990	Observations for SO_2_ and smoke obtained from a single monitoring station	Surgical confirmation of SOM	No statistically significant correlations between air pollutant levels and SOM hospital admissions					None

## Supplementary Materials

The following are available online at http://www.mdpi.com/1660-4601/15/2/257/s1, Figure S1: Funnel plot for the studies investigating associations between birth or first year of life NO2 exposure and subsequent otitis media in children; Table S1: Study quality using Newcastle-Ottawa Quality Assessment Scale for cohort studies; Table S2: Exposure to NO_2_, PM_2.5_, PM_2.5_ absorbance or PM_10_ and otitis media in children—summary results of cohort studies; Table S3: Study quality using Newcastle-Ottawa Quality Assessment Scale for case-control and case-crossover studies; Table S4: Study quality of time series studies using quality assessment tool adapted from Zaza et al. (Maximum score in brackets); Table S5: Study quality using Newcastle-Ottawa Quality Assessment Scale for cross-sectional studies.
