# Supplementary material for: Air Pollution and Otitis Media in Children: A Systematic Review of Literature
Source: Int J Environ Res Public Health. 2018 Feb 3;15(2):257. doi: 10.3390/ijerph15020257 (PMC5858326; doi:10.3390/ijerph15020257)
Supplement: Supplementary file 1 [file ijerph-15-00257-s001.pdf]

Supplementary Materials: Air Pollution and Otitis Media in Children: A Systematic Review of Literature

Summary of Assessed Quality of Included Studies

Table S1. Study quality using Newcastle-Ottawa Quality Assessment Scale for cohort studies.

Study Name and the Author	Representativeness	Selection of Non-Exposed Cohorts	Ascertainment of Exposure	Outcome of Interest not Present at Start	Comparability †	Assessment of Outcome	Adequate Follow up Time	Adequate Follow up of Cohorts	Score/10
Aguilera, Pedersen [1])	*	*	*	*	**	-	*	*	8
Brauer, Gehring [2]	*	*	*	*	**	-	*	*	8
Deng, Lu [3])	*	*	-	*	**	-	*	*	7
Deng, Lu [4])	*	*	-	*	**	-	*	*	7
Dostal, Prucha [5])	*	*	-	*	*	*	*	*	7
Jedrychowski, Galas [6])	*	*	*	*	**	-	*	*	8
MacIntyre, Gehring [7])	*	*	*	*	**	**	*	*	9
Brauer, Karr [8])	*	*	*	*	**	*	*	*	9
Pettigrew, Gent [9]	*	*	-	*	**	*	-	-	6

† Key confounders identified: socio-economic status and maternal smoking.

Table S2. Exposure to NO_2, $PM_{2.5}$, $PM_{2.5}$ absorbance or PM_{10} and otitis media in children – summary results of cohort studies.

Study and Sample Size	Exposure Window	Level of Exposure NO_2	OR/RR and 95% CI NO_2	Level of Exposure $PM_{2.5}$	OR/RR and 95% CI $PM_{2.5}$	Level of Exposure $PM_{2.5}$ Absorbance	OR/RR and 95% CI $PM_{2.5}$ Absorbance	Level of Exposure PM_{10} Absorbance	OR/RR and 95% CI PM_{10} Absorbance
Aguilera, Pedersen (1) INMA *n* = 2199 *	Entire prenatal	10 µg/m³	1.18 (0.98, 1.41)	N/A	N/A	N/A		N/A	N/A
	1st trimester	10 µg/m³	1.11 (0.99, 1.24)	N/A	N/A	N/A		N/A	N/A
	2nd trimester	10 µg/m³	1.16 (0.98, 1.37)	N/A	N/A	N/A		N/A	N/A
	3rd trimester	10 µg/m³	1.12 (0.98, 1.29)	N/A	N/A	N/A		N/A	N/A
	1st year of life	10 µg/m³	1.15 (1.01, 1.31)	N/A	N/A	N/A		N/A	N/A
Brauer, Gehring (2) PIAMA	1st year of life	3 µg/m³	1.17 (1.03,1.34)	10 µg/m³	1.13 (0.98, 1.32)	0.5 µg/m³	1.11 (0.98, 1.26)	N/A	N/A
n = 2984 (1 st year) *n* = 2970 (2 nd year)	1st 2 years of life	3 µg/m³	1.14 (1.03,1.27)	10 µg/m³	1.13 (1.00, 1.27)	0.5 µg/m³	1.10 (1.00, 1.22)	N/A	N/A
Brauer, Gehring (2) LISA	1st year of life	3 µg/m³	1.09 (0.78,1.54)	10 µg/m³	1.19 (0.73, 1.92)	0.5 µg/m³	1.12 (0.83, 1.51)	N/A	N/A
n = 620 (1 st year) *n* = 605 (2 nd year)	1st 2 years of life	3 µg/m³	1.14 (0.87,1.49)	10 µg/m³	1.24 (0.84, 1.83)	0.5 µg/m³	1.10 (0.86, 1.41)	N/A	N/A
Deng, Lu (3) *n* = 1617	prenatal	12 µg/m³	1.10 (0.76, 1.60)	N/A	N/A	N/A	N/A	7 µg/m³	0.95 (0.73, 1.23)
	postnatal	13 µg/m³	1.16 (0.73, 1.83)	N/A	N/A	N/A	N/A	6 µg/m³	1.36 (0.95, 1.94)
Deng, Lu (4) *n* = 1617	1st trimester	17 µg/m³	0.89 (0.57, 1.37)	N/A	N/A	N/A	N/A	15 µg/m³	0.91 (0.67, 1.26)
	2nd trimester	15 µg/m³	1.20 (0.83, 1.74)	N/A	N/A	N/A	N/A	14 µg/m³	1.03 (0.77, 1.39)
	3rd trimester	14 µg/m³	1.10 (0.77, 1.56)	N/A	N/A	N/A	N/A	16 µg/m³	0.89 (0.65, 1.22)
MacIntyre, Karr (10) British Colombia *n* = 45,513	During birth	10 µg/m³	1.09 (1.07, 1.12)	1.8 µg/m³	0.91 (0.89, 0.93)	1.1 µg/m³	0.94 (0.93, 0.96)	N/A	N/A
MacIntyre, Gehring (7)									
BAMSE, *n* = 3,821	During birth	10 µg/m³	1.01 (0.81, 1.27)	5 µg/m³	0.95 (0.66, 1.38)	1 unit	1.19 (0.63, 2.21)	10 µg/m³	0.94 (0.78, 1.14)
GASPII, *n* = 678	During birth	10 µg/m³	1.02 (0.84, 1.23)	5 µg/m³	0.75 (0.46, 1.22)	1 unit	0.79 (0.55, 1.12)	10 µg/m³	0.84 (0.59, 1.2)
INMA Asturias, *n* = 360	During birth	10 µg/m³	1.1 (0.89, 1.36)	N/A	N/A	N/A	N/A	N/A	N/A
INMA Gipuzkoa, *n* = 437	During birth	10 µg/m³	1.64 (1.03, 2.63)	N/A	N/A	N/A	N/A	N/A	N/A
INMA Sabadell, *n* = 402	During birth	10 µg/m³	0.94 (0.76, 1.15)	5 µg/m³	0.8 (0.34, 1.87)	1 unit	0.87 (0.53, 1.43)	10 µg/m³	1.06 (0.56, 1.98)
INMA Valencia, *n* = 559	During birth	10 µg/m³	1.13 (0.95, 1.34)	N/A	N/A	N/A	N/A	N/A	N/A
LISA South, *n* = 3,321	During birth	10 µg/m³	1.05 (0.86, 1.29)	5 µg/m³	1.06 (0.58, 1.94)	1 unit	1.08 (0.63, 1.87)	10 µg/m³	1.23 (0.73, 2.07)
LISA North, *n* = 2,460	During birth	10 µg/m³	0.9 (0.33, 2.44)	5 µg/m³	0.85 (0.13, 5.42)	1 unit	2.47 (0.51, 12)	10 µg/m³	1.21 (0.11, 14)
PIAMA, *n* = 3,475	During birth	10 µg/m³	1.15 (1.03, 1.29)	5 µg/m³	2.06 (1.25, 3.39)	1 unit	1.42 (1.06, 1.91)	10 µg/m³	1.61 (0.86, 3.01)
Combined	During birth	10 µg/m³	1.09 (1.02, 1.16)	5 µg/m³	1.06 (0.75, 1.49)	1 unit	1.08 (0.83, 1.39)	10 µg/m³	0.98 (0.84, 1.14)

Table S3. Study quality using Newcastle-Ottawa Quality Assessment Scale for case-control and case-crossover studies.

Study Name and the Author	Adequate Case Definition	Representative ness of Cases	Selection of Controls	Definition of Controls	Comparability †	Assessment of Exposure	Method of Ascertainment	Nonresponse Rate	Score/10
Daigler, Markello [11] ‡	*	-	*	*	-	-	*	*	5
da Costa, Navarro [12] ‡	*	*	*	*	-	-	*	*	5
Xiao, Liu [13] #	*	*	*	*	**	*	*	*	9
Kousha and Castner[14] #	*	*	*	*	**	*	*	*	9
Strickland, Hao [15] #	*	*	*	*	**	*	*	*	9
Zemek, Szyszkowicz [16] #	*	*	*	*	**	*	*	*	7

† Key confounders identified: socio-economic status and maternal smoking

Table S4. Study quality of time series studies using quality assessment tool adapted from Zaza et al (Maximum score in brackets).

Study Name and the Author	Study Design (7)	Population Eligibility and Sampling Described (4)	Valid and Reliable Exposure Measures (2)	Valid and Reliable Outcome Measures (2)	Appropriate Statistical Methods (3)	Multilevel Analyses (2)	Interpretation (1)	Confounding and Bias Addressed (3)	Score/23
Gestro, Condemi [17]	2	4	2	2	3	2	1	3	19

Table S5. Study quality using Newcastle-Ottawa Quality Assessment Scale for cross-sectional studies.

Study Name and the Author	Representativeness	Sample Size	Non-Respondents	Ascertainment of Exposure	Comparability †	Assessment of Outcome	Statistical Test	Score/10
Harvey [18]	*	-	-	-	-	**	-	3
Bhattacharyya and Shapiro [19]	*	*	-	*	-	*	*	5
Bhopal, Phillimore [20]	*	*	-	*	-	-	*	4
Heinrich, Hoelscher [21]	*	*	-	*	**	-	*	6
Heinrich, Hoelscher [22]	*	*	-	*	**	-	*	6
Sprem and Branica [23]	-	-	-	*	-	**	-	3
Ribeiro and Cardoso [24]	*	-	*	*	-	-	*	4
Holtby, Elliott [25]	*	*	*	-	-	*	-	4

† Key confounders identified: socio-economic status and maternal smoking

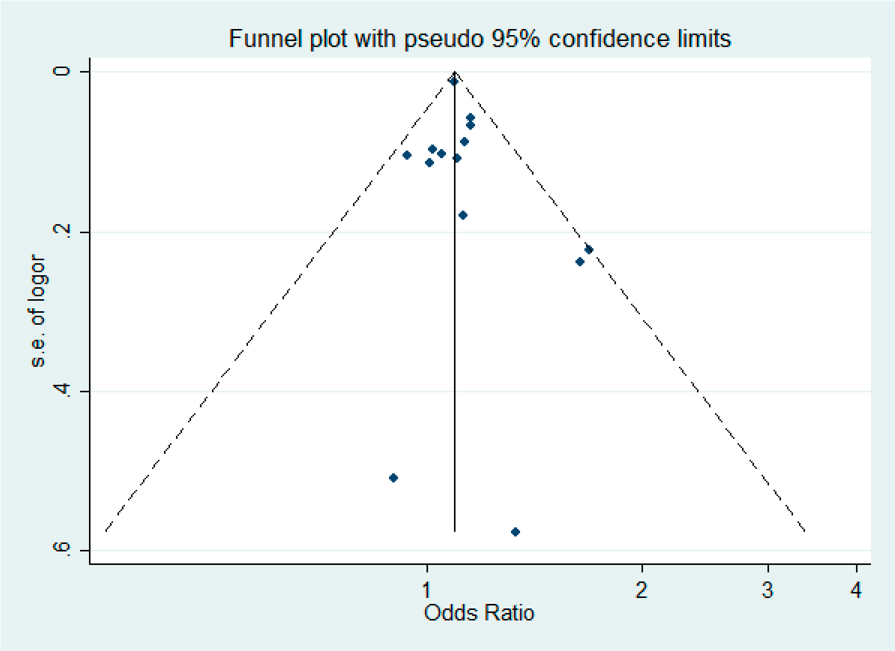

Figure S1. Funnel plot for the studies investigating associations between birth or first year of life NO_2 exposure and subsequent otitis media in children.

References

1. Aguilera, I.; Pedersen, M.; Garcia-Esteban, R.; Ballester, F.; Basterrechea, M.; Esplugues, A.; Fernández-Somoano, A.; Lertxundi, A.; Tardón, A.; Sunyer, J. Early-life exposure to outdoor air pollution and respiratory health, ear infections, and eczema in infants from the INMA study. *Environ. Health Perspect.* **2013**, *121*, 387–392.
2. Brauer, M.; Gehring, U.; Brunekreef, B.; de Jongste, J.; Gerritsen, J.; Rovers, M.; Wichmann, H.E.; Wijga, A.; Heinrich, J. Traffic-related air pollution and otitis media. *Environ. Health Perspect.* **2006**, *114*, 1414–1418.
3. Deng, Q.; Lu, C.; Jiang, W.; Zhao, J.; Deng, L.; Xiang, Y. Association of outdoor air pollution and indoor renovation with early childhood ear infection in China. *Chemosphere* **2017**, *169*, 288–296.
4. Deng, Q.; Lu, C.; Li, Y.; Chen, L.; He, Y.; Sundell, J.; Norbäck, D. Association between prenatal exposure to industrial air pollution and onset of early childhood ear infection in China. *Atmos. Environ.* **2017**, *157*, 18–26.
5. Dostal, M.; Prucha, M.; Rychlikova, E.; Pastorkova, A.; Sram, R.J. Differences between the spectra of respiratory illnesses in children living in urban and rural environments. *Cent. Eur. J. Public Health* **2014**, *22*, 3–11.
6. Jedrychowski, W.; Galas, A.; Pac, A.; Flak, E.; Camman, D.; Rauh, V.; Perera, F. Prenatal ambient air exposure to polycyclic aromatic hydrocarbons and the occurrence of respiratory symptoms over the first year of life. *Eur. J. Epidemiol.* **2005**, *20*, 775–782.
7. MacIntyre, E.A.; Gehring, U.; Mölter, A.; Fuertes, E.; Klümper, C.; Krämer, U.; Quass, U.; Hoffmann, B.; Gascon, M.; Brunekreef, B.; et al. Air pollution and respiratory infections during early childhood: An analysis of 10 European birth cohorts within the ESCAPE project. *Environ. Health Perspect.* **2014**, *122*, 107–113.
8. Brauer, M.; Karr, C.; MacIntyre, E.; Clark, N.; Su, J.; Baribeau, A.M.; Tamburic, L.; Lencar, C.; Demers, P.; Buzzelli, M.; et al. Woodsmoke and children's health: Findings from the border air quality study. *Epidemiology* **2011**, *22*, S186.
9. Pettigrew, M.M.; Gent, J.F.; Triche, E.W.; Belanger, K.D.; Bracken, M.B.; Leaderer, B.P. Infant otitis media and the use of secondary heating sources. *Epidemiology* **2004**, *15*, 13–20.
10. MacIntyre, E.A.; Karr, C.J.; Koehoorn, M.; Demers, P.A.; Tamburic, L.; Lencar, C.; Brauer, M. Residential air pollution and otitis media during the first two years of life. *Epidemiology* **2011**, *22*, 81–89.
11. Daigler, G.E.; Markello, S.J.; Cummings, K.M. The effect of indoor air pollutants on otitis media and asthma in children. *Laryngoscope* **1991**, *101*, 293–296.
12. da Costa, J.L.; Navarro, A.; Neves, J.B.; Martin, M. Household wood and charcoal smoke increases risk of otitis media in childhood in Maputo. *Int. J. Epidemiol.* **2004**, *33*, 573–578.

13. Xiao, Q.; Liu, Y.; Mulholland, J.A.; Russell, A.G.; Darrow, L.A.; Tolbert, P.E.; Strickland, M.J. Pediatric emergency department visits and ambient Air pollution in the U.S. State of Georgia: A case-crossover study. *Environ. Health Glob. Access Sci. Source* **2016**, *15*, 1–8.
14. Kousha, T.; Castner, J. The Air Quality Health Index and Emergency Department Visits for Otitis Media. *J. Nurs. Scholarsh. Off. Publ. Sigma Theta Tau Int. Honor Soc. Nurs. Sigma Theta Tau* **2016**, *48*, 163–171.
15. Strickland, M.J.; Hao, H.; Hu, X.; Chang, H.H.; Darrow, L.A.; Liu, Y. Pediatric Emergency Visits and Short-Term Changes in PM2.5 Concentrations in the U.S. State of Georgia. *Environ. Health Perspect.* **2016**, *124*, 690–696.
16. Zemek, R.; Szyszkowicz, M.; Rowe, B.H. Air pollution and emergency department visits for otitis media: A case-crossover study in edmonton, Canada. *Environ. Health Perspect.* **2010**, *118*, 1631–1636.
17. Gestro, M.; Condemi, V.; Bardi, L.; Fantino, C.; Solimene, U. Meteorological factors, air pollutants, and emergency department visits for otitis media: A time series study. Int. J. Biometeorol. 2017, 61, 1749–1764.
18. Harvey, R.M. Environmental factors in glue ear. *J. Laryngol. Otol*. **1975**, *89*, 73–77.
19. Bhattacharyya, N.; Shapiro, N.L. Air quality improvement and the prevalence of frequent ear infections in children. *Otolaryngol. Head Neck Surg.* **2010**, *142*, 242–246.
20. Bhopal, R.S.; Phillimore, P.; Moffatt, S.; Foy, C. Is living near a coking works harmful to health? A study of industrial air pollution. *J. Epidemiol. Community Health* **1994**, *48*, 237–247.
21. Heinrich, J.; Hoelscher, B.; Frye, C.; Meyer, I.; Pitz, M.; Cyrys, J.; Wjst, M.; Neas, L.; Wichmann, H.E. Improved air quality in reunified Germany and decreases in respiratory symptoms. *Epidemiology* **2002**, *13*, 394–401.
22. Heinrich, J.; Hoelscher, B.; Wichmann, H.E. Decline of ambient air pollution and respiratory symptoms in children. *Am. J. Respir. Crit. Care Med.* **2000**, *161*, 1930–1936.
23. Sprem, N.; Branica, S. Effects of sulphur dioxide and smoke on the incidence of secretory otitis media. *Arh. Hig. Rada Toksikol.* **1993**, *44*, 229–232.
24. Ribeiro, H.; Cardoso, M.R.A. Air pollution and children's health in São Paulo (1986–1998). *Soc. Sci. Med.* **2003**, *57*, 2013–2022.
25. Holtby, I.; Elliott, K.; Kumar, U. Is there a relationship between proximity to industry and the occurrence of otitis media with effusion in school entrant children? *Public Health* **1997**, *111*, 89–91.

© 2018 by the authors. Submitted for possible open access publication under the terms and conditions of the Creative Commons Attribution (CC BY) license (http://creativecommons.org/licenses/by/4.0/).
